# Immunogenetic Variation and Differential Pathogen Exposure in Free-Ranging Cheetahs across Namibian Farmlands

**DOI:** 10.1371/journal.pone.0049129

**Published:** 2012-11-07

**Authors:** Aines Castro-Prieto, Bettina Wachter, Joerg Melzheimer, Susanne Thalwitzer, Heribert Hofer, Simone Sommer

**Affiliations:** 1 Evolutionary Genetics Research Group, Leibniz Institute for Zoo and Wildlife Research, Berlin, Germany; 2 Evolutionary Ecology Research Group, Leibniz Institute for Zoo and Wildlife Research, Berlin, Germany; University of California, Berkeley, United States of America

## Abstract

**Background:**

Genes under selection provide ecologically important information useful for conservation issues. Major histocompatibility complex (MHC) class I and II genes are essential for the immune defence against pathogens from intracellular (e.g. viruses) and extracellular (e.g. helminths) origins, respectively. Serosurvey studies in Namibian cheetahs (*Acinonyx juabuts*) revealed higher exposure to viral pathogens in individuals from north-central than east-central regions. Here we examined whether the observed differences in exposure to viruses influence the patterns of genetic variation and differentiation at MHC loci in 88 free-ranging Namibian cheetahs.

**Methodology/Principal Findings:**

Genetic variation at MHC I and II loci was assessed through single-stranded conformation polymorphism (SSCP) analysis and sequencing. While the overall allelic diversity did not differ, we observed a high genetic differentiation at MHC class I loci between cheetahs from north-central and east-central Namibia. No such differentiation in MHC class II and neutral markers were found.

**Conclusions/Significance:**

Our results suggest that MHC class I variation mirrors the variation in selection pressure imposed by viruses in free-ranging cheetahs across Namibian farmland. This is of high significance for future management and conservation programs of this species.

## Introduction

Genetic variation at adaptive gene loci provides direct information on selective processes involving the interaction of individuals with their environment and their capacity for future adaptive changes [1], [2]. Particularly relevant are genes of the Major Histocompatibility Complex (MHC) which evolve under selection pressure imposed by pathogens [2], [3], [4]. MHC genes encode MHC class I (MHC I) and class II (MHC II) proteins that recognize and present pathogen-derived peptides from intracellular (e.g. viruses) and extracellular (e.g. bacteria, helminths) origins, respectively, to cytotoxic and T-helper cells, thereby triggering a cascade of immune responses in vertebrates [5]. Two main selection scenarios are currently debated to explain susceptibility or resistance to parasite infections. One scenario relies on the resistance effects of specific MHC alleles selected frequency-dependent in space and time in form of *negative frequency dependent selection*, also known as *rare-allele advantage* [6], [7] or in terms of *fluctuating selection* [8], [9]. The other scenario is based on the assumption that two MHC molecules with different binding properties present different subsets and thus a higher variety of antigens. Heterozygous individuals (*heterozygote advantage, overdominance* [10]), or individuals carrying highly dissimilar alleles (*divergent-allele advantage* [10], [11]) are capable of initiating an appropriate immune response against a more diverse array of pathogens than homozygous individuals or individual with more similar alleles. These non-exclusive hypotheses have been studied by testing associations of specific MHC alleles with pathogens, the proportions of genotypes within populations, patterns of population structure, and the effects of genetic distance between genotypes [2], [12]. The majority of these studies focused on MHC II genotyping and simultaneously scanned for different types of pathogens of extracellular origin in the same individuals [12], [13]. In contrast to these studies, we present a novel approach that also permits a retrospective analysis. We conducted MHC I and II analysis in a carnivore population for which serological data were available from previous studies and formulated predictions on MHC allele/haplotype frequencies, heterozygosity, and genetic distance for the two selection scenarios.

For this study, we focused onthere is the world's largest remaining population of free-ranging African cheetahs (*Acinonyx jubatus*). This population occurs in Namibia with an estimated population size of 3,100 to 5,800 individuals [14] and is considered a key population for the long-term survival of the species [15]. Free-ranging Namibian cheetahs concentrate in central Namibia, where a large proportion of individuals inhabit unprotected areas that encompass continuous privately owned livestock or game farmland often bordering towns and cities [16]. Consequently, free-ranging cheetahs on Namibian farmland potentially come into close proximity with domestic and feral dogs and cats that are sometimes unvaccinated [17]. Feral dogs and cats can be important vectors of diseases such as canine distemper, which is known to have affected free-ranging cheetahs in the Serengeti, Tanzania [18], [19]. Serosurveys of common feline and canine viruses such as feline coronavirus (FCoV), feline herpesvirus 1 (FHV1), feline calicivirus (FCV), feline parvovirus (FPV) and canine distemper virus (CDV) confirmed the exposure of free-ranging and captive Namibian cheetahs to these viral pathogens and a similar virus diversity across the country, but no evidence of clinical signs of infectious diseases in the examined individuals was detected [20], [21]. However, results from these studies showed substantial differences in the exposure to viral pathogens between free-ranging Namibian cheetahs from north-central [20] and east-central [21] regions. The proportion of cheetahs tested positive for viruses was higher in north-central than in east-central Namibia [20], [21]. In north-central Namibia 65.3%, 48.0%, 29.2%, 24.3% and 12.2% of the cheetahs were seropositive for FCV, FPV, FCoV, CDV and FHV1, respectively [20], whereas in east-central Namibia only 4.5% (*P*<0.0001), 3.0% (*P*<0.0001), 3.0% (*P*<0.0001), 4.5% (*P* = 0.0012) and 3.0% (*P* = 0.059) of the cheetahs were seropositive for these viruses [21]. This result was attributed to higher human population densities in the north-central than in the east-central region which would affect contact opportunities of cheetahs with (non-vaccinated) domestic and feral cats and dogs [21].

There is no genetic differentiation at evolutionarily neutral microsatellite markers among cheetahs throughout Namibia [22] indicating that this is a panmictic population and gene flow between individuals of north-central and east-central regions exists. Neutral genetic variation provides information on the demographic and evolutionary history of natural populations, whereas adaptive genetic variation provides information on selective processes involving the interaction of individuals with their environment and their capacity for future adaptive changes [2]. Therefore, patterns of neutral and adaptive variation may differ markedly in free-ranging populations [23].

In this study, we investigated variation in adaptive immune genes in functionally important regions of MHC I and II in cheetahs from north-central and east-central Namibia known from previous observations to differ substantially in their viral pathogen exposure [20], [21] and test predictions on the patterns of MHC variation within the current population under the two major different selection scenarios. An essential pre-requisite for testing these predictions is the locus-specific assignment of MHC alleles in the species, which was previously conducted in Namibian cheetahs by combining information on the evolutionary affinities between the observed MHC alleles, qualitative expression analysis, and the distribution of alleles among 149 individuals [24]. A total of ten MHC I alleles and four MHC II-DRB alleles were identified in the population [24]. Here we compared patterns of MHC I and II-DRB exon 2 variation in terms of allelic diversity, heterozygosity and genetic distance among genotypes, and estimated the levels of genetic differentiation using allele and haplotype frequencies between free-ranging Namibian cheetahs from regions that differ in viral pathogen exposure. The heterozygote or divergent allele advantage hypothesis predicts a higher heterozygosity and higher genetic distance at MHC I loci in cheetahs from north-central than east-central Namibia due to an increased exposure to viruses in north-central Namibia. The higher selection pressure in this region might also lead to a higher allelic diversity.

However, if specific MHC alleles are selected in a frequency-dependent manner in space and time according to their functional importance, we expect differences in the allele and haplotype frequencies between the two regions. Owing to missing information on extracellular pathogen distribution in Namibia, we have no clear prediction for MHC II-DRB. No differences in MHC II genetic variation would indicate a uniform extracellular pathogen distribution or suggest that intracellular-derived pathogens exert a stronger selection pressure than extracellular-derived pathogens. Results from this study will contribute to a better understanding of the conservation implications of MHC variation and differential pathogenic selection pressures on the Namibian cheetah population, which may be of great interest to wildlife management when relocation of individuals for conservation purposes is deliberated.

## Results

### Overall genetic diversity pattern

A total of six and four nucleotide sequences corresponding to MHC I exon 2 (229 bp) and MHC II-DRB exon 2 (246 bp) alleles, respectively, were observed in 88 cheetahs from Namibia. All alleles have been previously reported for the Namibian cheetah population and are known to be expressed (Genbank accession numbers AJU07665-66, GU971407, GU971409, GU971411, GU971414, AY312960-63 [24], [25], [26]). We previously reported 10 MHC I alleles, which is the consequence of the analysis of a longer sequence, i.e. exon 2-intron 2-exon 3) [24]. In the present study, we focused on exon 2 encoding the functionally important antigen-binding sites (ABS), i.e. amino acid positions postulated to interact directly with the foreign antigens [27], [28].

Based on the SSCP banding pattern it was not possible to discriminate between MHC I alleles *AJUMHCAJUI1* and Acju-MHCI*04 which differ only in a single amino acid position and refer to them as Acju-MHCI*04^§^ in this study. The substitution is outside the functionally important antigen-binding sites and does not affect the results of this study in any way. We previously determined the locus-specific assignment of MHC alleles in *A. jubatus* [24]. Using this information, we identified in this study seven and three genotypes for the six and four expressed alleles at the MHC I and II-DRB loci, respectively (Table 1). Locus A at MHC I and loci A and B at MHC II were monomorphic in all individuals analyzed, and thus they are likely to represent fixed alleles in the population, whereas the loci B and D at MHC I and locus C at MHC II were polymorphic. Allele and haplotype frequencies from polymorphic class I (MHC I-B, MHC I-D) and class II (MHC II DRB-C) loci are presented in Table 2. Mean H_E_ was 0.35±0.21 across both polymorphic MHC I loci and 0.50 for the polymorphic MHC II-DRB locus. This is lower than the mean expected heterozygosity observed at 38 microsatellite loci (mean H_E_ = 0.64–0.71; [12]). H-W equilibrium was met at locus C of MHC II DRB but it deviated significantly (*P* = 0.0004) across loci B and D of MHC I, resulting in a deficit of heterozygotes (Table 2). This is due to the locus B (*P* = 0.005) because locus D remained at H-W equilibrium (*P* = 0.30) (data not in Table 2).

**Table 1:** MHC class I exon 2 and class II-DRB exon 2 genotypes detected in Namibian cheetahs.

MHC I	Locus A	Locus B		Locus D		No. Indiv.
	*AJUMHC AJUI3*	Acju-MHCI*04§	Acju-MHCI*02	Acju-MHC*05	Acju-MHC*07	
I	X	X	X	X		28 (32%)
II	X	X	X	X	X	2 (2%)
III	X		X	X		14 (16%)
IV	X		X	X	X	10 (11%)
V	X	X		X		28 (32%)
VI	X	X		X	X	4 (5%)
VII	X	X			X	2 (2%)

MHC II	Locus A	Locus B		Locus C		
	AcjuFLA-DRB1*ha17	AcjuFLA-DRB1*ha16		AcjuFLA- DRB1*ha14	AcjuFLA-DRB1*ha15	
I	X	X		X		17 (19%)
II	X	X		X	X	50 (57%)
III	X	X			X	21 (24%)

X indicates presence of a given allele; § refers to either allele Acju-MHCI*04 or *AJUMHCAJUI1*. Genbank accession numbers for each allele are given in parenthesis as follows: *AJUMHCAJUI3* (U07666), Acju-MHCI*02 (GU971407), Acju-MHCI*04§ (GU971408 or U07665), Acju-MHCI*05 (GU971409), Acju-MHCI*07 (GU971411), AcjuFLA-DRB1*ha14 (AY312960), AcjuFLA-DRB1*ha15 (AY312961), AcjuFLA-DRB1*ha16 (AY312962), and AcjuFLA-DRB1*ha17 (AY312963).

**Table 2:** Genetic variation and differentiation at MHC class I (a) and class II-DRB (b) genes in Namibian cheetahs.

	All	Region			Sex		
		East-central		North-central	Males		Females
**MHC class I exon 2 (locus B & D)**							
**Sample size (** ***N*** **)**	88	62		26	67		21
**Haplotype frequencies**							
Acju-MHCI*04^§^/Acju-MHCI*05	0,51	0,58		0,35	0,49		0,6
Acju-MHCI*02/Acju-MHCI*05	0,38	0,32		0,52	0,39		0,33
Acju-MHCI*02/Acju-MHCI*07	0,07	0,06		0,1	0,07		0,05
Acju-MHCI*04^§^/Acju-MHCI*07	0,05	0,05		0,04	0,05		0,02
**Hardy-Weinberg equilibrium**							
Mean *H_O_*/*H_E_*	0.26±0.11/	0.25±0.10/		0.29±0.14/	0.26±0.09/		0.26±0.30/
	0.35±0.21	0.33±0.20		0.36±0.17	0.36±0.20		0.31±0.25
Exact *P*	***	*		ns	**		ns
**Genetic differentiation**							
*F_ST_* across loci			0,07			−0.001	
*P* (*F_ST_*)			**			ns	
Exact *P* (differentiation test)			*			ns	
χ^2^			9,19			1,84	
*df*			2			2	
*P* (χ^2^)			**			ns	
*P* (Fisher)			*			ns	
**MHC class II exon 2 (locus C)**							
**Sample size (** ***N*** **)**	88	62		21	62		26
**Allele frequencies**							
AcjuFLA-DRB1*ha14	0,48	0,47		0,5	0,49		0,43
AcjuFLA-DRB1*ha15	0,52	0,53		0,5	0,51		0,57
**Hardy-Weinberg equilibrium**							
*H_O_*/*H_E_*	0.57/0.50	0.55/0.50		0.61/0.51	0.63/0.50		0.38/0.50
Exact *P*	ns	ns		ns	ns		ns
**Genetic differentiation**							
*F_ST_*			−0			−0,01	
*P* (*F_ST_*)			ns			ns	
Exact *P* (differentiation test)			ns			ns	
*P* (Fisher)			ns			ns	

Only polymorphic MHC loci were included in these analyses.

* P≤0.05,

** P≤0.01,

*** P≤0.001, ns not significant. § refers to either allele Acju-MHCI*04 or *AJUMHCAJUI1*.

### Comparisons between cheetahs in north-central and east-central Namibia

Allelic diversity was identical in both regions; all observed MHC I and II-DRB alleles were present in north-central and east-central Namibia. Measures of MHC genetic variation and genetic differentiation between north-central and east-central Namibian cheetahs are summarized in Table 2. Although levels of heterozygosity across MHC I loci were similar in cheetahs from north-central (mean H_E_ = 0.36±0.17) and east-central (mean H_E_ = 0.33±0.20) regions, a slight heterozygosity deficit compared to H-W expectations was observed across MHC I loci in east-central cheetahs. The amino acid distance of MHC I haplotypes did not differ between regions (Mann-Whitney U-Test, mean distance_north-central_ = 18.46±7.69, mean distance_east-central_ = 15.79±7.11, *P* = 0.14). Levels of heterozygosity at the MHC II-DRB locus were also similar in both regions and no deviation from H-W equilibrium was detected.

**Figure 1:**
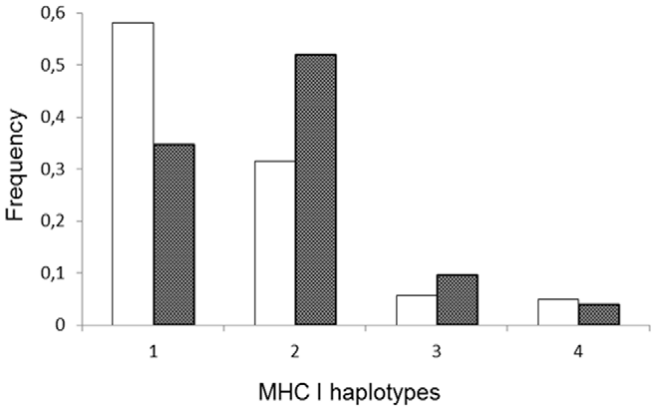
Haplotype frequencies at MHC class I (exon 2) loci between north-central and east-central Namibian cheetahs.

In contrast, patterns of genetic differentiation in MHC I and MHC II-DRB loci differed between the two regions (Table 2). Namibian cheetahs from the north-central region were highly differentiated from those of the east-central region based on the haplotype frequencies across MHC I loci (F_ST_ = 0.07, p<0.01). The differentiation remained significant even after re-assigning north-central individuals trapped close to the region borders (*N* = 7) to the east-central region (F_ST_ = 0.03, p = 0.03) or after excluding them from the analysis (F_ST_ = 0.05, p = 0.04). This result was mainly driven by a large difference in the haplotype frequencies of Acju-MHCI*04^§^/Acju-MHCI*05 and *Acju*-MHCI*02/Acju-MHCI*05 between individuals from north-central and east-central Namibia (Fig. 1). In contrast to the MHC I loci, no differentiation at the MHC II-DRB locus (F_ST_ = −0.01, p = 0.74) was detected between regions. Exact tests of differentiation also revealed significant differences between the regions based on the haplotype frequencies across MHC I loci (p = 0.02) but not on the allele frequencies at MHC II-DRB locus (p = 0.61). MHC I differentiation between both regions was further supported by combining a chi-square test (χ^2^ = 9.19, df = 2, p = 0.01) and Fisher's exact test (p = 0.02) in a locus-by-locus treatment.

### Comparisons between male and female cheetahs in Namibia

All MHC I and II-DRB alleles were present in both sexes. Levels of observed heterozygosity across MHC I loci were identical in males and females (H_obs_ = 0.26). The expected heterozygosity was slightly higher in males (mean H_E_ = 0.36±0.20) than in females (mean H_E_ = 0.31±0.25) and deviated from H-W expectations (Table 2). At the MHC II-DRB locus, the observed heterozygosity was significantly higher in males (H_obs_ = 0.63) than in females (H_obs_ = 0.38, χ^2^ = 3.94, p<0.05) but no deviation from H-W equilibrium was detected. No genetic differentiation at any locus of MHC I or MHC II-DRB was detected between male and female Namibian cheetahs (Table 2).

## Discussion

Two main selection scenarios are currently debated to explain susceptibility or resistance to pathogen infections. They focus either on the effects of (1) specific MHC alleles which are selected in a frequency-dependent manner in space and time, or (2) a heterozygote or divergent allele advantage. The heterozygote or divergent allele advantage hypothesis assumes that heterozygous or genetically more diverse individuals are able to respond to a larger range of pathogen peptides than homozygotes and, consequently, benefit from increased resistance to pathogens. Owing to a higher selective pressure imposed by viruses in north-central than east-central Namibia, we expected to detect a higher allelic diversity, heterozygosity and genetic distance at MHC I loci. However, cheetahs from both regions showed similar levels of genetic variation in terms of allelic diversity, heterozygosity and amino acid distance at MHC I loci. Thus, we did not find any evidence for a heterozygote or divergent allele advantage. The genetic variation at MHC II DRB loci was also similar in both regions.

Whereas seroprevalence data reflect the percentage of the population exposed to certain pathogens, it does not reflect the effects of multiple infections in individuals or differences in pathogen diversity in a population. However, it may be the diversity of viral pathogens or short-term shifts in virus pressures that cause a heterozygote advantage. Diverse and even pleiotropic interactions of pathogens and potential specialist and generalist MHC alleles in terms of different pathogen detection might co-exist on the individual level and thus obscure the conformity on the population level.

We detected, however, clear differences in the haplotype frequencies across MHC I loci, resulting in a significant differentiation between cheetahs from north-central and east-central regions. This implies that specific MHC I alleles/haplotypes were selected in a frequency-dependent manner in space and time according to their functional importance by either *negative frequency dependent selection*, *rare-allele advantage* [6], [7] or *fluctuating selection* [8], [9]. Fluctuating selection, i.e. spatial and temporal heterogeneity in the abundance of pathogens, could well cause significant differences in allele and haplotype frequencies across populations even if the overall allelic diversity remains constant. While individuals from these regions were highly differentiated at the MHC I loci, no difference was observed at MHC II-DRB locus.

The two serology studies on which our predictions were based used different protocols for some of the tests [20], [21]. However, it is unlikely that this affected the results in a significant way. As argued by Thalwitzer et al. [21], the use of different protocols is only likely to lead to different results if the investigated virus is highly variable in antigenicity [29], [30], as is likely the case with for example FCV and FCoV [31], [32], but not if it is antigenetically conserved, as is likely with FHV, FPV and CDV [33], [34], [35]. Thalwitzer et al. [21] further argued that serum neutralisation tests are more specific and thus more conservative than immunofluorescence assay tests. This is because the former tests detect antibodies only when they bind to relatively small areas on the viral surface, whereas the latter tests detect antibodies directed to a broader array of epitopes on the viral surface [36]. Because of the antigene conservatism in some of the viruses and because the antibodies to several viruses in north-central Namibian cheetahs were tested with serum neutralisation tests [20], [21], the difference between the higher seroprevalence in north-central and the lower seroprevalence in east-central Namibian cheetahs for FHV, FPV, CDV, and FCV is likely to be an underestimate and thus reflect genuine differences in seroprevalence.

In epidemiological terms, the higher seroprevalences to viral pathogens observed in north-central than in east-central Namibia was attributed to the higher human density there and therefore also a higher (non-vaccinated) feral cat and dog density in north-central Namibia, an important factor that could influence transmission opportunities for pathogens [21]. Because MHC I fights against intracellular-derived pathogens such as viruses and MHC II-DRB against extracellular-derived pathogens such as bacteria and helminths, the contrasting distribution of genetic variation between MHC I and MHC II-DRB loci suggests that intracellular-derived pathogens exert a stronger selection pressure than extracellular-derived pathogens in cheetahs across Namibian farmland. Alternatively, the lack of MHC II-DRB differentiation in Namibian cheetah may also be explained by extra-cellular pathogens exerting strong selective pressure that is spatially uniform across Namibia. In order to further understand and draw conclusions on the pathogen-mediated mechanisms that act on MHC II-DRB variation of the Namibian cheetah population, data on extracellular-derived pathogens such as their diversity, abundance and distribution is essential.

We also examined the influence of male-biased sampling on the patterns of MHC variation observed in the population and did not detect any differences between males and females on the allele and haplotype frequencies at any MHC loci. This is consistent with both sexes being similarly exposed to most viruses of concern to cheetahs [20]. As there was no obvious sex-specific difference, we think it is unlikely that the larger number of males in this study affected any of our conclusions.

Demographic processes are predicted to affect all loci, whereas selective processes are not expected to influence neutrally evolving loci. Therefore, the effects of variation in population size and other historical processes become visible on the genetic level by comparing different types of molecular markers [4]. To control for the confounding effects of demographic processes on patterns of MHC variation in the Namibian cheetah population, we compared the extent of genetic differentiation at both MHC classes with the level estimated for neutral microsatellites by Marker et al. [22]. The latter study revealed 248 alleles in 38 microsatellite loci from 89 free-ranging Namibian cheetahs originating from the same regions as in the present study. A lack of genetic differentiation at the microsatellites (mean F_ST_ = 0.02, P>0.05) among regions indicates that free-ranging Namibian cheetahs represent a large panmictic population [22]. Hence, the patterns of genetic differentiation at adaptive MHC I and neutral microsatellites differed markedly in the Namibian cheetah population. The high genetic differentiation observed in MHC I but not in microsatellites between Namibian cheetahs from north-central and east-central regions is therefore a likely consequence of selection pressure driven by viral pathogens rather than demographic processes affecting gene flow. Therefore, genetic differentiation at MHC I loci of the contemporary Namibian cheetah population appears to be maintained by an ongoing selective process. It is difficult, however, to draw conclusions based solely on empirical comparisons between MHC genes and microsatellites because the mutational processes and selective regimes of these genetic markers are different [37]. Still, we showed with this study that information on seroprevalences from previous studies can be used to test predictions derived from different selection scenarios and to make conclusions on the pathogen-mediated mechanisms acting on a free-ranging population.

### Conservation implications

Fitness-related genes of adaptive significance such as those of the MHC are crucial for the long-term conservation of a species in the wild, and therefore of primary interest in conservation genetics [37], [38]. Conservation plans based solely on neutral genetic variation are not guaranteed to preserve adaptive genetic variation because both sources of genetic variation are not always positively correlated. Gene flow within a population might result in low differentiation in neutral markers, but there might still be high differentiation in adaptive markers in the population [37]. The Namibian cheetah population represents a large panmictic population as revealed by neutral microsatellite markers [22]. Yet, our results show a high differentiation in adaptive MHC I loci between cheetahs from east-central and north-central regions. This finding is of great relevance for the conservation of the Namibian cheetah population. Translocation of cheetahs within Namibia is an increasingly common management tool for conservation purposes. In light of our results, such translocations should be carefully planned as a wrongly chosen origin of or destination area for translocated animals may adversely affect their ability to cope with different pathogenic selection pressures. Therefore, further research should focus on MHC composition in relation to pathogen load in cheetahs from different African populations.

## Materials and Methods

### Ethics statement

Our study on free-ranging and captive cheetahs was approved by the Namibian Ministry of Environment and Tourism (MET) (permit number 1514/2011), the scientific advisory board of the AfriCat Foundation in Namibia and the Ethical Committee of the Leibniz Institute for Zoo and Wildlife Research in Berlin (permit number 2012-02-01). AfriCat Foundation is registered by the MET as a large non-profit carnivore captive facility since 1993 (permit office 2004/11) and runs a rehabilitation centre for free-ranging carnivores, mainly cheetahs. AfriCat is equipped with high standard veterinary facilities and qualified staff caring for the animals.

### Study site and sample collection

**Figure 2:**
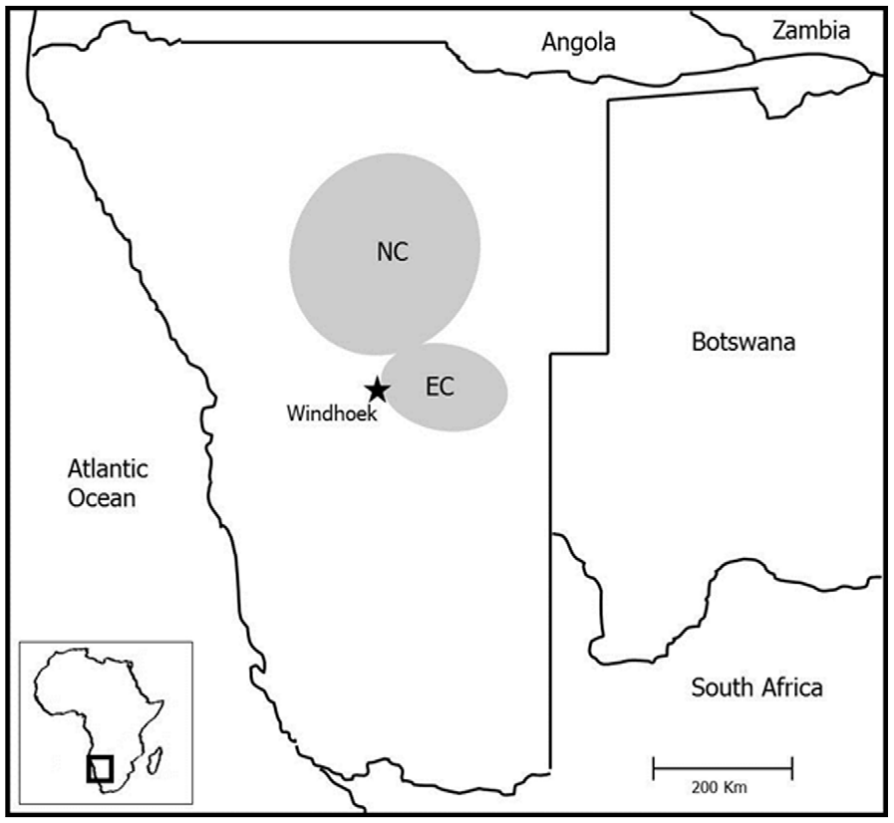
Schematic map showing the location (shaded) of the two study areas in Namibia from which cheetah samples derived.

We used blood samples from 88 wild-born cheetahs (67 males and 21 females) from east-central and north-central Namibia (Fig. 2). These animals included free-ranging individuals inhabiting commercial livestock or game farmland in Namibia (*N* = 49), wild-caught individuals kept in a large holding facility (*N* = 28) or dead individuals reported to us or found in the field (*N* = 11). For the MHC analyses, the captive cheetahs were allocated to the east-central or north-central region according to their place of capture. Free-ranging cheetahs were trapped near marking trees and immobilized as described in [39]. Blood samples were centrifuged and the buffy coat stored in liquid nitrogen. Samples were transported in a dry shipper in full compliance with the Convention on International Trade in Endangered Species (CITES) to Germany and stored at −80°C until further processing at the laboratory.

### Molecular methods

All 88 samples were genotyped for MHC I and II-DRB loci. Sixty-two individuals were already included in a previous study [24]. Total genomic DNA was isolated from blood buffy coat using the DNeasy Blood and Tissue Kit (Qiagen, Hilden, Germany) following the manufacturer's instructions. We focused our analyses on the second exons of MHC I and II because this region is highly polymorphic and includes functionally important antigen-binding sites (ABS), i.e. amino acid positions postulated to interact directly with foreign antigens [27], [28]. The second exon (229 bp) of MHC I alleles was amplified using the primer Acju_Ex2MhcI_cF (5′-GCTCCCACTCCCTGAGGTAT-3′; [23]) and the newly designed primer Acju_Ex2MhcI_kR (5′-GGAKTCGCTCTGGTTGTAGT-3′) designed from MHC I transcript sequences available from felid species in GenBank, following PCR conditions as described in Castro-Prieto *et al.* (2011). The second exon (246 bp) of MHC II-DRB alleles was amplified using primers and PCR conditions as described in Castro-Prieto *et al.* (2011). Genotyping of MHC I and II-DRB was conducted through single-stranded conformation polymorphism (SSCP) analysis followed by sequence analysis of the distinctive single-strand bands as previously described [24]. To ensure that the sequences represented true alleles, the PCR-SSCP analysis was conducted twice per individual sample.

### Data analysis

To examine patterns of sequence variation, nucleotide sequences were edited manually based on their forward and reverse consensus chromatograms using Chromas Pro Version 1.33 (Technelysium Pty Ltd). The sequences were aligned and coding regions were translated into deduced amino acid sequences using Clustal W as implemented in MEGA 3.1 [40]. The MHC-like nature of the sequences was verified through a homology analysis using blastn (http://blast.ncbi.nlm.nih.gov/Blast.cgi). Standard diversity indices were estimated using the software Arlequin 3.1 [41]. Allele frequencies were estimated at all putative loci separately and as a whole haplotype for MHC I loci. For haplotypes, the frequencies of co-occurring alleles (which presumably constitute a haplotype on a given chromosome) were estimated as the number of individual occurrences of a certain allele divided by its total count observed in the population. Expected heterozygosity (H_E_) was estimated as a general indicator of the amount of genetic variation in the population [42]. Departures from Hardy-Weinberg (H-W) equilibrium were assessed by applying exact tests [43]. The genetic distance between individual alleles were calculated by the number of amino acid substitutions using the Poisson model as implemented in MEGA 3.1 [40].

For the analysis of genetic variation and genetic differentiation at MHC I and II-DRB loci, all samples were classified into north-central or east-central region according to Thalwitzer *et al.* [21] (Fig. 2). These two regions lack physical barriers but differ substantially in viral pathogen exposure as revealed by seroprevalence studies conducted in Namibian cheetahs [20], [21]. The ratio of males and females was similar in the north-central (*N*_males_ = 18, *N*_females_ = 8) and the east-central region (*N*_males_ = 49, *N*_females_ = 13, χ^2^ = 0.97, df = 1, p = 0.33). The prediction of different allele and haplotype frequencies between regions was tested by using F-statistics [44] and exact tests of sample differentiation [45] as implemented in Arlequin 3.1. Additionally, we used the chi-square (χ^2^) and Fisher's method of combined P values obtained by Fisher's exact test as implemented in CHIFISH [46] for MHC I loci. This was done because we combined information from multiple loci which may result in low statistical power and prevent detection of true genetic divergence [47]. This problem occurs particularly in small contingency tables (few populations and few alleles per locus) as observed in our data set. We also tested for differences in the allele and haplotype frequencies between male and female cheetahs to control for any effect due to the male-biased sampling. Differences in heterozygosity between north-central and east-central Namibian cheetahs as well as between males and females were tested with chi-square test in SPSS Version 16.0.
